# Comparison of Spaeth/Richman Contrast Sensitivity and Pelli-Robson Tests for Assessing Contrast Sensitivity in Patients with Glaucoma

**DOI:** 10.18502/jovr.v20.16610

**Published:** 2025-12-08

**Authors:** Fatema Noble, Suhas Haldipurkar, Vijay Shetty, Tanvi Haldipurkar, Rita Dhamankar, Devendra Venkatramani, Shreyas Dhamorikar, Sarita Deshpande, Maninder Singh Setia

**Affiliations:** Laxmi Eye Hospital and Laxmi Charitable Trust, Panvel, Maharashtra, India

**Keywords:** Contrast Sensitivity, Diagnostic Test Properties, Glaucoma, Pelli-Robson, Spaeth/Richman Contrast Sensitivity Test

## Abstract

**Purpose:**

To determine the optimal cut-offs for Pelli-Robson (PR) and Spaeth/Richman contrast sensitivity (SPARCS) test scores for diagnosing glaucoma and to compare PR and SPARCS scores (total and individual quadrants) for assessing contrast sensitivity in patients with glaucoma.

**Methods:**

This study was a single-center, cross-sectional, two-group analysis of 87 glaucomatous eyes and 87 non-glaucomatous control eyes. We assessed visual acuity, refraction, intraocular pressure (IOP), cup disc ratio (CDR), and anterior chamber depth in these patients. The PR score for central contrast sensitivity was obtained, and the SPARCS scores were generated for four outer zones and the central region.

**Results:**

The mean IOP [SD] was significantly higher in the glaucoma group (19.3 [5.2] mm Hg) compared with the control group (17.5 [3.6] mm Hg; *P *= 0.008). The mean CDR [SD] was significantly higher in the glaucoma group compared with the control group (0.73 [0.14] vs. 0.46 [0.12]; *P* < 0.001). The mean [SD] PR score (1.48 [0.17] vs. 1.23 [0.19]; *P* < 0.001) and total SPARCS score (78.2 [5.1] vs. 62.4 [11.2]; *P* < 0.001) were significantly higher in the control group compared with the glaucoma group. The optimal cut-off for identifying glaucoma was 1.35 for the PR score and 70 for the total SPARCS score. At this value of SPARCS score, the sensitivity for identifying glaucoma was 83.9% (95% CI, 74.5 to 90.9), specificity was 96.6% (95% CI, 90.3 to 99.3), positive predictive value (PPV) was 96.1% (95% CI, 88.9 to 99.2), and negative predictive value (NPV) was 85.7% (95% CI, 77.2 to 92.0). The area under the curve (AUC) value was significantly higher for the total SPARCS score compared with the PR score (0.92 vs. 0.83; *P *= 0.001). All individual SPARCS scores (superior nasal, superior temporal, central, inferior nasal, and inferior temporal) had lower AUC, sensitivity, specificity, PPV, and NPV values compared with the total SPARCS score.

**Conclusion:**

At the optimal cut-offs, the total SPARCS score offers significantly better diagnostic test properties for identifying glaucoma compared with the PR score.

##  INTRODUCTION

Contrast sensitivity (CS) provides information about functional vision—a feature which is usually not captured by visual acuity or visual field.^[1,2]^ Campbell and Green were among the first to report diminished CS in patients with open-angle glaucoma.^[3]^ It has also been reported that about 30% of optic nerve axons are lost before visual defects appear in patients with glaucoma.^[4,5]^ Thus, there was a need to identify and investigate the specific retinal changes due to glaucoma.^[6,7]^ The Pelli-Robson (PR) chart has been shown to be an effective, consistent, and reliable tool for assessing spatial CS in patients with glaucoma.^[8]^ However, the PR chart evaluates only central CS, which may not accurately reflect visual function in patients with glaucoma who have peripheral arcuate field defects.^[1,9,10,11]^

To address some of these concerns, the relatively newer test called Spaeth/Richman contrast sensitivity (SPARCS) was designed to detect CS in patients with glaucoma.^[12]^ SPARCS is an online testing method that assesses CS in four peripheral quadrants—which correspond to typical glaucomatous visual field defects—as well as one central quadrant.^[1,11,12,13,14,15,16]^ The test uses square wave gratings rather than letters and is relatively easy to use; besides, it is not expensive and may not require an elaborate set-up.^[11,12,15]^ In this test, the patient must identify the quadrant in which the gratings are displayed, which implies it may also be used in patients with low literacy levels. Previous studies have shown that SPARCS has good repeatability and is sensitive and specific in identifying patients with glaucoma.^[17]^ Other studies have found this test to be repeatable even in individuals with refractive errors, with no significant difference between various types of errors.^[13]^ In addition, the test may help stage glaucoma and assess visual function in these patients.^[11]^

As indicated earlier, the SPARCS test assesses CS in each of the peripheral quadrants (upper lateral, upper medial, lower lateral, lower medial) and the central quadrant. In this study, we aimed to identify the optimal cut-offs for the score for each quadrant as well as the total SPARCS score. In addition, we compared the PR chart and SPARCS scores (total and individual quadrants) to assess CS in patients with glaucoma.

##  METHODS

The present study is a cross-sectional analysis of 174 eyes of 108 patients. Of these, 87 eyes from 50 patients were in the glaucoma group, and 87 eyes from 58 patients were in the control (non-glaucomatous) group.

### Study Site and Participants

The study was conducted at Laxmi Eye Institute and Laxmi Charitable Trust, Panvel, India, from September 2021 to August 2022. It is a tertiary eye care center with all specialties. We included patients older than 40 years of age who were able to provide informed consent, and they were classified as cases if diagnosed with primary open-angle glaucoma (POAG). The criteria for POAG included an open angle on gonioscopy, along with glaucomatous changes in the optic nerve head (ONH) and retinal nerve fiber layer (RNFL), which are typically associated with characteristic glaucomatous visual field defects. A trained glaucoma specialist performed gonioscopy, fundus examination, visual field testing, and RNFL assessment. The Anderson criteria for visual field were used to diagnose the disease and to monitor disease progression. If the patients did not meet the aforementioned criteria for glaucoma, we classified them as controls (non-glaucomatous group). We excluded patients who had undergone incisional eye surgery in the past 3 months or laser therapy in the past 1 month. Other patients who were excluded were those with best-corrected visual acuity < 20/200 and those with visually significant cataract or visual impairment due to ocular or neurological diseases other than glaucoma that would affect CS. We had 39 cases of pseudophakia in the present analysis, all of which had a monofocal intraocular lens (IOL). In addition, we did not include any patients with multifocal IOLs—as this may alter CS—or any with pre-perimetric glaucoma.

### Study Procedures

All patients were evaluated clinically. A comprehensive ocular evaluation was performed that included:

• Corrected distance visual acuity (CDVA), using Snellen and logMAR (logarithm of the minimum angle of resolution) chart;
• IOP assessed by Goldmann applanation tonometry;
• Detailed anterior segment examination, using slit lamp biomicroscopy, and a meticulous evaluation of the anterior chamber angle with an indentation gonioprism, to exclude angle-closure glaucoma or other secondary causes of increased IOP;
• Posterior segment evaluation was performed using a 90-diopter (D) lens;
• Optic nerve evaluation, using the Disc Damage Likelihood Scale based on the rim-to-disc ratio (staging from 1 to 10) and the cup disc ratio (CDR).

The visual field defects were assessed with Humphrey Field Analyzer (24-2 Swedish Interactive Threshold Algorithm Standard Strategy; Carl Zeiss Meditec, Inc., Oberkochen, Germany) using Anderson's criteria.^[14]^ CS was evaluated using the PR and SPARCS tests, as described below.

### Pelli-Robson test

The PR test evaluates CS in the central region and consists of a large wall-mounted chart with a luminance setting of 85 cd/m². It consists of 16 triplets of Sloan letters of constant size arranged in eight horizontal lines. The contrast gradually drops by 0.15 log units from 100% (0.00 log units) to 0.56% (2.25 log units) after every three letters. Participants were asked to read the letters. When an individual failed to correctly identify two of the three letters in a triplet, the test was terminated, and their log CS score was recorded.^[15]^

### Spaeth/Richman contrast sensitivity test (SPARCS)

SPARCS is a computer-based assessment that can be conducted on any computer that has internet connectivity and is available at https://www.sparcscontrastcenter.com. Every patient is assigned a unique identification number after following the standard testing protocol provided by the website. Participants were examined in a dimly lit room with undilated pupils while sitting at a distance of 50 centimeters from the computer screen. At this distance, the test assessed 30º of horizontal and 23.5º of vertical vision, while the central test area covered 5º horizontally and 3.5º vertically. Patients were told to focus on the central area of the testing screen and determine the portions that appeared different. Then, they would temporarily lose fixation in order to identify the area showing the grating. The individuals would then re-fixate on the central area and click it to reveal the next image. To assess the contrast threshold, the examiner would show the patient vertical square-wave gratings with a spatial frequency of 0.4 cycles per degree that occurred for 0.3 seconds in one of the five examined locations. The contrast level was advanced by four levels for each correct response until the patient gave an incorrect response. At this point, the contrast level would decrease by two levels. Following that, the algorithm was advanced or regressed by one level at a time until the patient gave two incorrect replies for one level. After this point, the threshold for that individual in this specific region was determined. The contrast range examined was 100 % to 45 % (Log CS, 0.00–2.35), with a drop of about 0.15 log units between levels. Each of the four outer zones, as well as the central area, received its own score. Each of the five areas contributed to a total SPARCS score, and the total score of all five areas (ideal sum) was 100.^[11,12,15]^

### Statistical Analysis

Data were entered in Excel (Microsoft Corp, Redmond, WA, USA) and analyzed using Stata Version 17 (StataCorp LLC, College Station, TX, USA).

We calculated the mean and standard deviations (SD) or median and interquartile range (IQR) for continuous variables, and proportions for categorical variables. The means were compared using the *t*-test, and the medians were compared using the Wilcoxon Mann-Whitney test. The proportions were compared using the chi-square test or Fisher's exact test for low expected cell counts. We estimated the Pearson's correlation coefficient between two linear variables. Then, we used the receiver operating characteristic (ROC) curve to estimate the area under the curve (AUC) and identify the optimal cut-offs for SPARCS scores. After identifying the cut-offs, we estimated the sensitivity, specificity, positive predictive value (PPV), and negative predictive value (NPV). We also classified the glaucoma group into mild/moderate/severe glaucoma.^[18]^ We compared the AUC for the total SPARCS score and PR score for mild, moderate, and severe glaucoma. We also assessed the optimal cut-off for differentiating between mild and moderate/severe glaucoma. In addition, regression models were used for multivariate analysis to estimate the independent association between the total SPARCS score and PR score. We used visual field index (VFI), IOP, CDR, anterior chamber depth (ACD), age, and gender as covariates in the multivariate regression model. A *P*-value of < 0.05 was considered statistically significant.

The study was approved by the Institutional Ethics Committee of Laxmi Eye Institute and Charitable Trust (Ref No. LEI/003/2021; January 21, 2022).

**Table 1:** Demographic and clinical characteristics of glaucomatous eyes and controls (Panvel, India)

**Parameter**	**Total**	**Glaucoma**	**Control**	***P*** **-value**
Total [textitn (%)]	174 (100)	87 (50)	87 (50)	
Age [mean (SD)]	56.3 (8.6)	55.4 (8.3)	57.2 (8.9)	0.17
Gender [textitn (%)]				
Male	106 (60.9)	53 (60.9)	34 (39.1)	–
Female	68 (39.1)	53 (60.9)	34 (39.1)	
Eye [textitn (%)]				
Right eye	91 (52.3)	43 (49.4)	44 (50.6)	0.45
Left eye	83 (47.7)	48 (55.2)	39 (44.8)	
Refraction [median (IQR]				
Spherical	1.0 (–0.5, 2.0)	1.0 (–1.0, 1.5)	1.75 (0.5, 2.25)	0.005
Cylinder	–0.75 (–1, –0.5)	–0.75 (–1, –0.5)	–0.5 (–1, –0.5)	0.53
Spherical equivalent	0 (–0.25, 1.0)	0 (–0.5, 0.75)	0 (–0.25, 1.0)	0.48
Add	2.5 (2.0, 2.5)	2.5 (2.0, 2.5)	2.5 (2.0, 2.5)	0.93
Vision				
CDVA [median (IQR]	0 (0, 0)	0 (0, 0)	0 (0, 0)	< 0.001
CNVA [textitn (%)]				
N6	157 (90.2)	70 (80.5)	87 (100)	< 0.001
N8/N10	13 (7.5)	13 (14.9)	0 (0)	
N12/N18	2 (1.2)	2 (2.3)	0 (0)	
< N18	2 (1.2)	2 (2.3)	0 (0)	
IOP [mean (SD)]	18.4 (4.6)	19.3 (5.2)	17.5 (3.6)	0.008
ACD [textitn (%)]				
Shallow	44 (25.3)	25 (28.7)	19 (21.8)	0.30
Deep	130 (74.7)	62 (71.3)	68 (78.2)	
Cup-to-disc ratio [mean (SD)]	0.60 (0.19)	0.73 (0.14)	0.46 (0.12)	< 0.001
MD [median (IQR]	–0.645 (–5.45, 0.65)	–5.08 (–12.99, 0.50)	0.45 (–1.01, 0.65)	< 0.001
SD, standard deviation; IQR, interquartile range; CDVA, corrected distance visual acuity; CNVA, corrected near visual acuity; IOP, intraocular pressure; ACD, anterior chamber depth; MD, mean deviation.				

**Table 2:** Mean and standard deviations for Pelli-Robson and SPARCS parameters (Panvel, India)

**Parameter**	**Total**	**Glaucoma**	**Control**	***P*** **-value**
Total [textitn (%)]	174 (100)	87 (50)	87 (50)	
Pelli-Robson [mean (SD)]	1.35 (0.22)	1.23 (0.19)	1.48 (0.17)	< 0.001
SPARCS [mean (SD)]				
Total	70.4 (11.8)	62.4 (11.2)	78.2 (5.1)	< 0.001
Superior temporal	15.1 (2.8)	13.8 (2.9)	16.5 (1.9)	< 0.001
Superior nasal	14.5 (3.2)	12.6 (3.2)	16.5 (1.8)	< 0.001
Inferior temporal	13.7 (3.1)	12.0 (3.3)	15.4 (1.7)	< 0.001
Inferior nasal	13.9 (2.9)	11.6 (3.1)	15.0 (1.5)	< 0.001
Central	13.6 (2.6)	12.5 (2.5)	14.7 (2.1)	< 0.001
SPARCS, Spaeth/Richman contrast sensitivity.				

**Table 3:** Diagnostic test properties of cut-offs for Pelli-Robson and SPARCS parameters

**Parameter**	**Cut-off value**	**AUC**	**Sensitivity (%)**	**Specificity (%)**	**PPV (%)**	**NPV (%)**
		**Estimate (95% CI)**	**Estimate (95% CI)**	**Estimate (95% CI)**	**Estimate (95% CI)**	**Estimate (95% CI)**
Pelli-Robson	1.35	0.72 (0.65 to 0.78)	83.9 (74.5 to 90.9)	59.8 (48.7 to 70.1)	67.6 (57.9 to 76.3)	78.8 (67.0 to 87.9)
SPARCS						
Total	70.0	0.90 (0.86 to 0.95)	83.9 (74.5 to 90.9)	96.6 (90.3 to 99.3)	96.1 (88.9 to 99.2)	85.7 (77.2 to 92.0)
Superior nasal	14.5	0.79 (0.74 to 0.85)	67.8 (56.9 to 77.4)	90.8 (82.7 to 95.9)	88.1 (77.8 to 94.7)	73.8 (64.4 to 81.9)
Superior temporal	14.5	0.71 (0.65 to 0.77)	52.9 (41.9 to 63.7)	89.7 (81.3 to 95.2)	83.6 (71.2 to 92.2)	65.5 (56.3 to 74.0)
Inferior nasal	14.0	0.79 (0.73 to 0.85)	72.4 (61.8 to 81.5)	85.1 (75.8 to 91.8)	82.9 (72.5 to 90.6)	75.5 (65.8 to 83.6)
Inferior temporal	14.0	0.79 (0.73 to 0.85)	66.7 (55.7 to 76.4)	90.8 (82.7 to 95.9)	87.9 (77.5 to 94.6)	73.1 (63.8 to 81.2)
Central	13.0	0.73 (0.67 to 0.79)	57.5 (46.4 to 68.0)	88.5 (79.9 to 94.3)	83.3 (71.5 to 91.7)	67.5 (58.1 to 76.0)
CI, confidence interval; AUC, area under the curve; PPV, positive predictive value; NPV, negative predictive value; SPARCS, Spaeth/Richman contrast sensitivity.						

**Figure 1:**
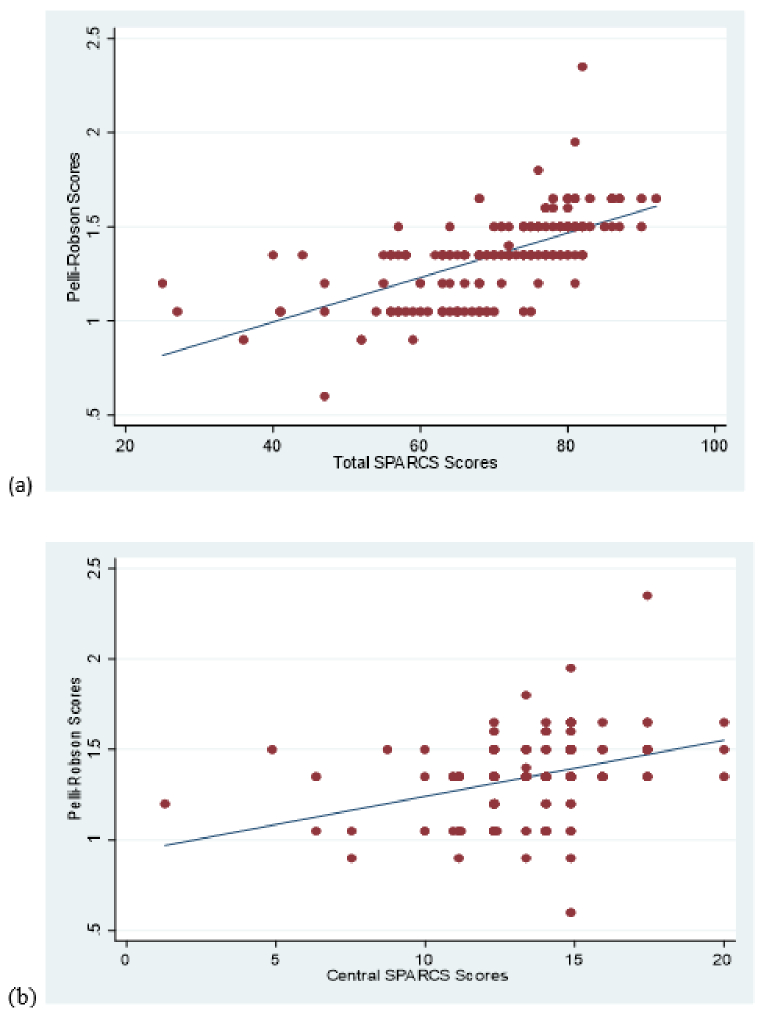
(a) Scatter plot for Pelli-Robson scores and total SPARCS scores. (b) Scatter plot for Pelli-Robson scores and central SPARCS scores. SPARCS, Spaeth/Richman contrast sensitivity.

**Figure 2:**
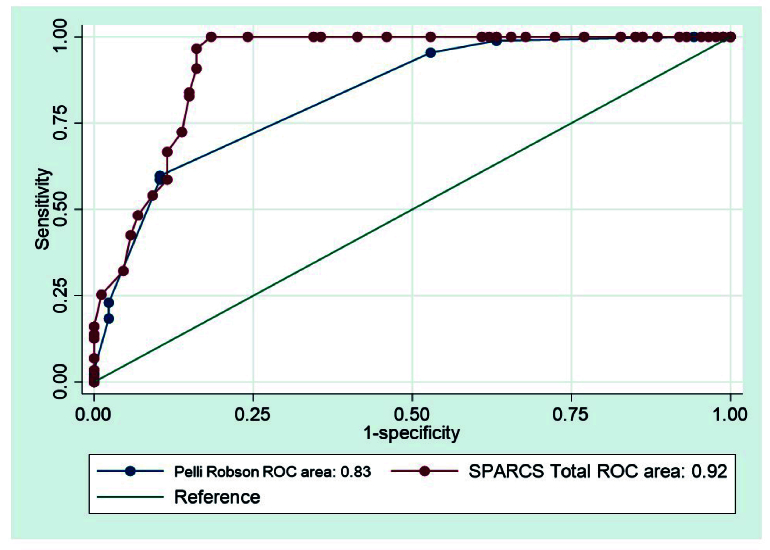
Comparison of Receiver Operating Characteristic curves for Pelli-Robson scores and total SPARCS scores in the identification of glaucoma. SPARCS, Spaeth/Richman contrast sensitivity.

**Figure 3:**
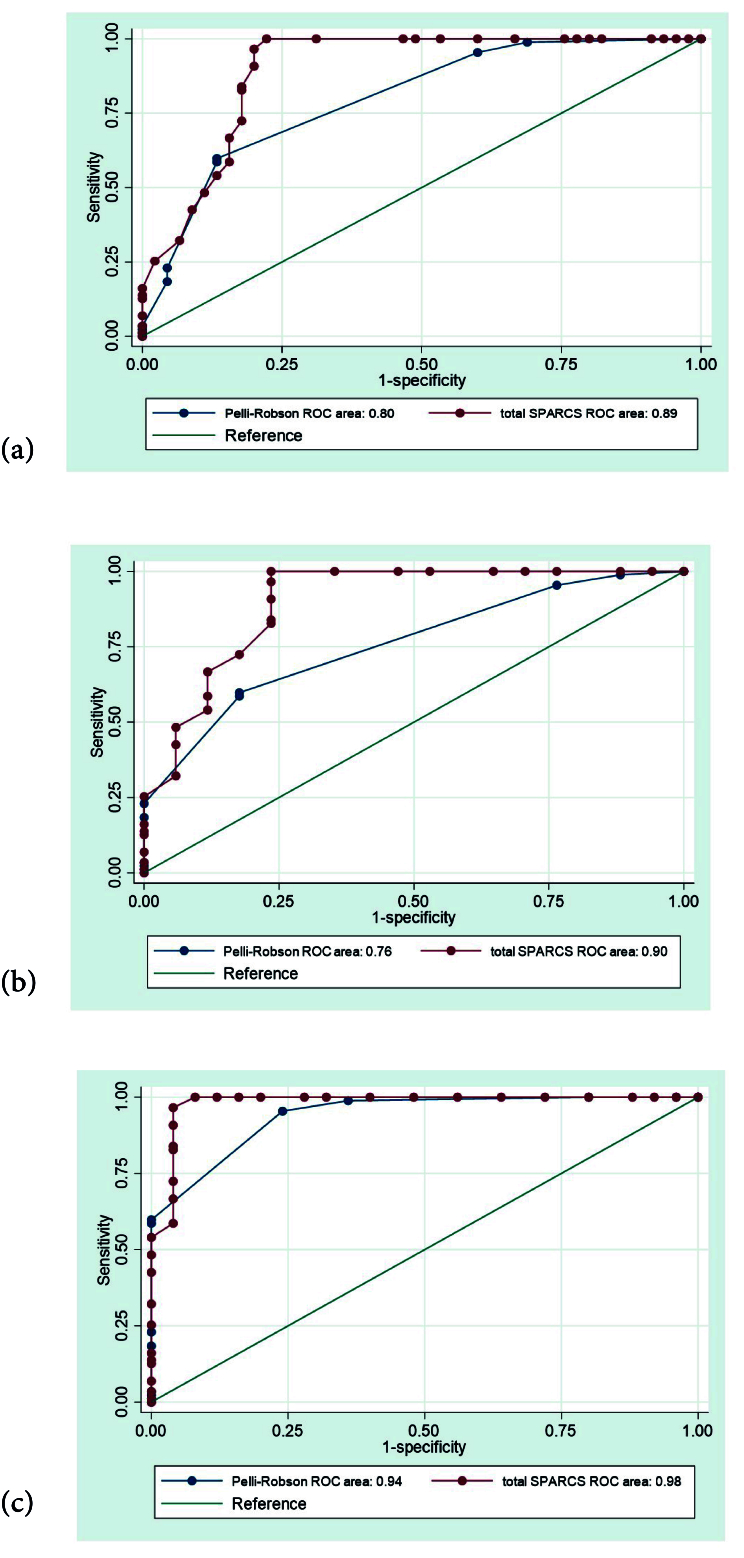
(a) Comparison of Receiver Operating Characteristic curves for Pelli-Robson scores and total SPARCS scores in the identification of mild glaucoma. (b) Comparison of ROC curves for Pelli-Robson scores and total SPARCS scores in identifying moderate glaucoma. (c) Comparison of ROC curves for Pelli-Robson scores and total SPARCS scores in identifying advanced/severe glaucoma. ROC, receiver operating characteristic; SPARCS, Spaeth/Richman contrast sensitivity.

##  RESULTS

The mean [SD] age was 55.4 [8.3] years in the glaucoma group and 57.2 [8.9] years in the control group (*P *= 0.17). Of the total population, 60.9% were male and 39.1% were female; this proportion was not significantly different across the glaucoma and control groups [Table 1]. The median [IQR] values for spherical refraction error were higher in the control group compared with the glaucoma group (1.75 [0.50, 2.25] vs. 1.0 –1.0, 1.5]; *P *= 0.005); however, there was no significant difference in spherical equivalent (0 [–0.25, 1.0] vs. 0 [–0.50, 0.75]; *P *= 0.48). A higher proportion of eyes in the control group had a CDVA of 6/6 on the Snellen acuity chart compared with the glaucoma group (97.7% vs. 75.9%; *P* < 0.001). The mean IOP [SD] was significantly higher in the glaucoma group (19.3 [5.2] mm Hg) compared with the control group (17.5 [3.6] mm Hg; *P *= 0.008). Similarly, the mean [SD] CDR was significantly higher in the glaucoma group compared with the control group (0.73 [0.14] vs. 0.46 [0.12]; *P* < 0.001). Detailed demographic and optic parameters for the glaucoma and control groups are presented in Table 1. The glaucoma group comprised 45 (51.7%) cases of mild glaucoma, 17 (19.5%) cases of moderate glaucoma, and 25 (28.7%) cases of advanced/severe glaucoma.

Table 2 presents the mean [SD] for PR and SPARCS scores. In general, the mean values were higher in the control group compared with the glaucoma groups. The mean [SD] PR score was significantly higher in the control group compared with the glaucoma group (1.48 [0.17] vs. 1.23 [0.19]; *P* < 0.001). Similarly, the mean [SD] total SPARCS score was significantly higher in the control group than in the glaucoma group (78.2 [5.1] vs. 62.4 [11.2]; *P* < 0.001). Pearson's correlation coefficient (*r*) was 0.64 (*P* < 0.001) between the PR score and total SPARCS score. This correlation was low between the PR score and the central SPARCS score (*r* = 0.36, *P* < 0.001) [Figures 1a & 1b], whereas it ranged from 0.52 to 0.54 for all other quadrants of the SPARCS scores. In the multivariate linear regression models, after adjusting for age, gender, VFI, IOP, CDR, and ACD, we found that each unit increase in the PR score was associated with an increase of 16.34 (95% CI, 9.74 to 22.95; *P* < 0.001) units in the total SPARCS score, suggesting a statistically significant association. Similarly, each unit increase in the PR score was associated with a change of 3.35 (95% CI, 1.25 to 5.45; *P* = 0.002) units in the SPARCS score for superior temporal region and an increase of 4.49 (95% CI, 2.30 to 6.69; *P* < 0.001) units in the SPARCS score for superior nasal region; both these associations were statistically significant. For the inferior region SPARCS score, we found that each unit increase in the PR score was associated with a change on 3.31 (95% CI, 1.17 to 5.45; *P* = 0.003) units in the SPARCS score for inferior temporal region and an increase of 2.91 (95% CI, 1.01 to 4.81; *P *= 0.003) units in the SPARCS score for inferior nasal region. These associations were also statistically significant.

The optimal cut-off for identifying glaucoma was 1.35 log units for the PR score; the AUC for this cut-off was 0.72 (95% CI, 0.65 to 0.78). At 1.35 log units, the sensitivity for identifying glaucoma was 83.9% (95% CI, 74.5 to 90.9), specificity was 59.8% (95% CI, 48.7 to 70.1), PPV was 67.6% (95% CI, 57.9 to 76.3), and NPV was 78.8% (95% CI, 67.0 to 87.9). The optimal cut-off for the total SPARCS score was 70.0. At this threshold, the sensitivity for identifying glaucoma was 83.9% (95% CI, 74.5 to 90.9), specificity was 96.6% (95% CI, 90.3 to 99.3), PPV was 96.1% (95% CI, 88.9 to 99.2%), and NPV was 85.7% (95% CI, 77.2 to 92.0). All individual SPARCS scores (superior nasal, superior temporal, central, inferior nasal, and inferior temporal) had lower AUC, sensitivity, specificity, PPV, and NPV values compared with total SPARCS scores [Table 3]. The AUC of the ROC curve was significantly higher for total SPARCS scores than for PR scores (*P *= 0.001) [Figure 2], indicating that total SPARCS scores were better at identifying glaucoma than PR scores. However, there was no significant difference between the PR score and SPARCS scores for each individual quadrant or the central value. Comparing the total SPARCS score with each individual quadrant score, we found that the total value was significantly better compared with all five quadrant values (*P* < 0.001). Among patients with glaucoma, the cut-off between mild and moderate/severe glaucoma was 60 for the total SPARCS score and 1.20 for the PR scores. However, the diagnostic test properties were not as good as the cut-off between the control and glaucoma groups. At the cut-off of 60 for total SPARCS score in differentiating between mild and moderate/severe glaucoma, the AUC was 0.65 (95% CI, 0.55 to 0.75), sensitivity was 50% (95% CI, 34.2 to 65.8), specificity was 80% (95% CI, 65.4 to 90.4), PPV was 70% (95% CI, 50.6 and 85.3), and NPV was 63.2% (95% CI, 49.3 and 75.6). At the cut-off of 1.20 for the PR score in differentiating between mild and moderate/severe glaucoma, the AUC was 0.56 (95% CI, 0.46 to 0.66), sensitivity was 42.9% (95% CI, 27.7 to 59.0), specificity was 68.9% (95% CI, 53.4 to 81.8), PPV was 56.3% (95% CI, 37.7 to 73.6), and NPV was 56.4% (95% CI, 42.3 to 69.7).

We also compared the AUC for the total SPARCS score and the PR score for each type of glaucoma. For mild glaucoma, the AUC for total SPARCS score (0.89, 95% CI, 0.82 to 0.96) was significantly higher compared with the AUC for PR score (0.80, 95% CI, 0.72 to 0.88; *P* < 0.001) [Figure 3a]. For moderate glaucoma, the AUC for total SPARCS score (0.90, 95% CI, 0.80 to 1.00) was significantly higher compared with the AUC for PR score (0.76, 95% CI, 0.66 to 0.86; *P *= 0.029) [Figure 3b]. In advanced/severe glaucoma, the AUC for total SPARCS score (0.98, 95% CI, 0.95 to 1.00) was higher compared with the AUC for PR scores (0.94, 95% CI, 0.90 to 0.98; *P *= 0.067) [Figure 3c].

##  DISCUSSION

We found that both PR and total SPARCS scores were significantly lower in the glaucoma group compared with the control group. The optimal cut-off for identifying glaucoma was 1.35 log units for the PR score and 70.0 for the total SPARCS scores. At this cut-off, SPARCS had significantly better diagnostic properties for identifying glaucoma compared with the PR score. However, there was no significant difference in the diagnostic test characteristics of PR score and SPARCS scores for the central quadrant or individual quadrants (superior nasal, superior temporal, inferior nasal, inferior temporal).

CS tests are helpful in diagnosing ocular conditions such as glaucoma.^[19]^ Although some authors have suggested that static CS is very useful to assess visual defect in patients with glaucoma, others have suggested that an isolated CS score may not be a good test in the absence of other associated clinical features.^[20,21]^ Nevertheless, it has been shown that a decreased CS is associated with visual field loss in patients with glaucoma and may be a good predictor for the performance of daily routines in these patients.^[1,22,23]^ Thus, assessing CS is important for diagnosis, prognosis, and management of patients with glaucoma.

In our study, despite the observed good correlation between the PR score and total SPARCS score, the latter was better at identifying glaucomatous eyes compared with the former. Furthermore, both scores were significantly lower in glaucomatous eyes compared with non-glaucomatous eyes. Both these findings have been reported by earlier studies as well. For instance, Thakur et al found a stronger correlation between these scores (0.79), whereas Sun et al found a lower correlation between them (0.44).^[13,24]^ The cut-offs identified in our study for both these scores were similar to those identified by Thakur et al. However, Thakur et al reported that sensitivity was slightly higher for the PR scores compared with SPARCS scores at a cut-off of 67 (84.4% vs. 70.0%).^[24]^ Other studies by Richman et al and Rao et al have also reported a similar sensitivity and specificity in identifying glaucoma and its severity.^[11,12]^ We compared the diagnostic properties of both tests statistically, and we did find that SPARCS was significantly better compared with PR scores at identifying glaucoma. Even for identifying mild and moderate glaucoma, the total SPARCS score was significantly better than PR scores. For advanced/severe glaucoma, although the AUC was higher for total SPARCS scores than for PR scores, the difference was not statistically significant (*P *= 0.067).

Furthermore, we estimated the diagnostic properties for each of the quadrants. The sensitivity, specificity, PPV, and NPV for SPARCS scores for individual quadrants were significantly lower than those of the total SPARCS score. Besides, the scores of these individual quadrants were not significantly different from the PR scores. Thus, we do not recommend using individual quadrant scores to assess CS in patients with glaucoma.

This was a one-time assessment of CS in patients with glaucoma compared with controls. A longitudinal assessment will help understand the progression of glaucoma. Ichhpujani et al studied the change in SPARCS scores over time and reported that these can be used to monitor the deterioration of glaucoma over time.^[25]^ As with other studies, we did analyze according to the severity of glaucoma.^[11]^ However, some authors have suggested that CS may not be a useful marker for glaucoma severity.^[26]^ Our primary focus was to compare the diagnostic test properties of SPARCS and PR and to understand the role of individual quadrant SPARCS scores in assessing CS in patients with glaucoma. Another limitation of our study was the lack of complete information on macular optical coherence tomography parameters for all patients.

Despite the limitations above, we found that SPARCS was significantly better than PR at measuring CS. The advantages of this test are that it has good reliability and it is not affected by the literacy status of the individual being tested;^[17,27]^ Thus, it may be useful in low-to-middle-income countries such as India, which has a large variability in literacy status across the country. Given the increasing reach of the internet in India, this may be a preferred method of CS assessment, even in remote and rural areas, as well as for community outreach programs, and will help in early detection of glaucoma and the initiation of treatment. We suggest adopting the total SPARCS score, as opposed to individual quadrant scores, as a marker of glaucoma.

##  Financial Support and Sponsorship

None.

##  Conflicts of Interest

None.
